# Detection of spondylosis deformans in thoracolumbar and lumbar lateral X-ray images of dogs using a deep learning network

**DOI:** 10.3389/fvets.2024.1334438

**Published:** 2024-02-15

**Authors:** Junseol Park, Hyunwoo Cho, Yewon Ji, Kichang Lee, Hakyoung Yoon

**Affiliations:** ^1^Department of Veterinary Medical Imaging, College of Veterinary Medicine, Jeonbuk National University, Iksan, Republic of Korea; ^2^Biosafety Research Institute and College of Veterinary Medicine, Jeonbuk National University, Iksan, Republic of Korea; ^3^Department of Electronic Engineering, Sogang University, Seoul, Republic of Korea

**Keywords:** disc instability, intervertebral disc space, artificial intelligence, bony spur, intervertebral disc disease, canine

## Abstract

**Introduction:**

Spondylosis deformans is a non-inflammatory osteophytic reaction that develops to re-establish the stability of weakened joints between intervertebral discs. However, assessing these changes using radiography is subjective and difficult. In human medicine, attempts have been made to use artificial intelligence to accurately diagnose difficult and ambiguous diseases in medical imaging. Deep learning, a form of artificial intelligence, is most commonly used in medical imaging data analysis. It is a technique that utilizes neural networks to self-learn and extract features from data to diagnose diseases. However, no deep learning model has been developed to detect vertebral diseases in canine thoracolumbar and lumbar lateral X-ray images. Therefore, this study aimed to establish a segmentation model that automatically recognizes the vertebral body and spondylosis deformans in the thoracolumbar and lumbar lateral radiographs of dogs.

**Methods:**

A total of 265 thoracolumbar and lumbar lateral radiographic images from 162 dogs were used to develop and evaluate the deep learning model based on the attention U-Net algorithm to segment the vertebral body and detect spondylosis deformans.

**Results:**

When comparing the ability of the deep learning model and veterinary clinicians to recognize spondylosis deformans in the test dataset, the kappa value was 0.839, indicating an almost perfect agreement.

**Conclusions:**

The deep learning model developed in this study is expected to automatically detect spondylosis deformans on thoracolumbar and lumbar lateral radiographs of dogs, helping to quickly and accurately identify unstable intervertebral disc space sites. Furthermore, the segmentation model developed in this study is expected to be useful for developing models that automatically recognize various vertebral and disc diseases.

## 1 Introduction

Spondylolysis deformans is a non-inflammatory degenerative change characterized by new bone formation in the endplates of the vertebral body (1). It is relatively common in dogs and can occur because of disc degeneration and corrected disc instability (2, 3). The exact mechanism remains unclear, but it is thought to be primarily due to the age-related destruction of peripheral annulus fibers (1, 3, 4). This can lead to discontinuity and weakening of disc attachment, stressing the ventral and dorsal longitudinal ligaments, which in turn can lead to herniation of the ventral or dorsal disc and development of spondylosis deformans (3). Previous studies have shown a correlation between intervertebral disc protrusion and the location of spondylosis deformans (2, 3, 5, 6). In addition, spondylosis deformans occurs on the dorsal aspect of the vertebrae and may progress to nerve root impingement or cause meningeal irritation, resulting in neurologic dysfunction (3).

Radiologically, spondylosis deformans is characterized by osteophyte formation in the vertebral endplate and can range from small bony proliferations of the vertebral endplate to the bridging of the adjacent vertebrae (1, 6, 7). In cases of obvious lesions, such as spondylosis deformans that form bridges, the diagnosis can be easily made on radiographs alone; however, if the lesions are very mild, the diagnosis can be somewhat subjective.

The use of deep learning models, a form of artificial intelligence, for the objective and accurate diagnosis of various disc and vertebral diseases is an increasingly active area of research in medicine and has recently shown promising results in various medical image analysis tasks such as classification, object detection, and segmentation (8–12). For image classification tasks, deep learning models can accurately detect diseases and abnormalities; however, they cannot accurately localize the exact regions of interest (ROI) (13, 14). Although object detection models can localize rough ROIs, semantic segmentation models offer the most accurate localization of abnormalities through pixel-wise classification (8, 15). Accurate localization of the ROI provided by segmentation models can be used to automatically measure parameters or detect abnormalities with interpretable results (16–18). In a recent medical study involving humans, a model was developed to automatically detect spondylolisthesis by accurately segmenting the lumbar spine in X-ray images (9). However, the use of deep learning models to diagnose vertebral diseases using the X-ray images of dogs has not been studied. In this study, we aimed to develop a novel deep learning-based segmentation model for thoracolumbar and lumbar lateral X-ray images of dogs for automatically recognizing vertebral bodies and detecting spondylosis deformans. Unlike previous studies, we focused on not only detecting the presence of spondylosis deformans but also accurately segmenting very small lesions, such as grade 1 spondylosis deformans, to large lesions, such as grade 3 spondylosis deformans. We also aimed to design a segmentation model that accurately separates the vertebral body and spondylosis deformans region with high accuracy by labeling each region accordingly pixel by pixel.

## 2 Materials and methods

### 2.1 Patient dataset

This retrospective study included patients who presented to Jeonbuk National University Animal Medical Center between April 2017 and October 2023 and underwent radiographic imaging of the thoracolumbar or lumbar vertebrae. In 162 dogs, X-ray images (ECO-BT-525 VET, EcoRay, Gwangju, Korea) were obtained and used to develop the deep learning models. Patients with and without specific clinical signs of disc disease were randomly selected and included in the study. For 152 of the 162 dogs, we conducted physical and neurologic examinations related to thoracolumbar and lumbar disc disease. We also investigated the relationship between spondylosis deformans and clinical signs associated with thoracolumbar and lumbar disc disease. Dogs were considered to have clinical signs if any of the following were identified: proprioceptive ataxia of the pelvic limbs, spinal pain, loss of the panniculus reflex, and loss of deep pain. This study was approved by the Institutional Animal Care and Use Committee of Jeonbuk National University (approval nos. JBNU NON2022-085 and NON2023-023).

### 2.2 Image dataset

#### 2.2.1 Radiographic image acquisition for deep learning model development

Spondylosis deformans typically initiates as a ventral or dorsal bony proliferation and extends adjacent to intervertebral discs to fill the gap. Consequently, it is more prominently visible on lateral X-ray images than on ventrodorsal or dorsoventral X-ray images. Hence, only lateral X-ray images were used in this study (1, 19). A total of 265 lateral thoracolumbar and lumbar lateral X-ray images (ECO-BT-525 VET; EcoRay, Gwangju, Korea) from 162 dogs were used for model development. For the training and validation datasets, we used images acquired under conditions of 66 kVp−70 kVp and 2.6 mAs−3.0 mAs. In thoracolumbar lateral X-ray images, the beam center was located at T12-T13 with a field of view (FOV) ranging from approximately T8 to L4, varying slightly between dogs. For lumbar X-ray images, the beam center was located at L3-L4, covering images from T12 to the cranial level of caudal vertebrae. All training and validation dataset images had the FOV set to best exclude abdominal organs, and these images were captured by keeping the vertebrae as straight as possible. The X-ray images used for the test dataset were plane abdominal lateral images acquired at 61 kVp−80 kVp and 10 mAs−14 mAs; these conditions depended on the size of the dog, as depicted by abdomen width measurements for each dog. The beam center was located around the last rib, the FOV was set to include all abdominal organs from the liver to the hips, and the images were cropped to the same area as the FOV of the training and validation datasets. All digital radiographic images were post-processed to maintain adequate contrast. Images containing motion artifacts and rotation of vertebrae in the acquired X-ray images were excluded from the study. The dataset distribution for training, validation, and test followed an approximate ratio of 80:10:10, and the training and validation data were chosen randomly. A total of 119 thoracolumbar lateral X-ray images (92 images for training data, 13 images for validation data, and 14 images for test data) and 146 lumbar lateral X-ray images (124 images for training data, 12 images for validation data, and 10 images for test data) were used to develop deep learning model.

#### 2.2.2 Evaluation of radiographic images

**Figure 1:**
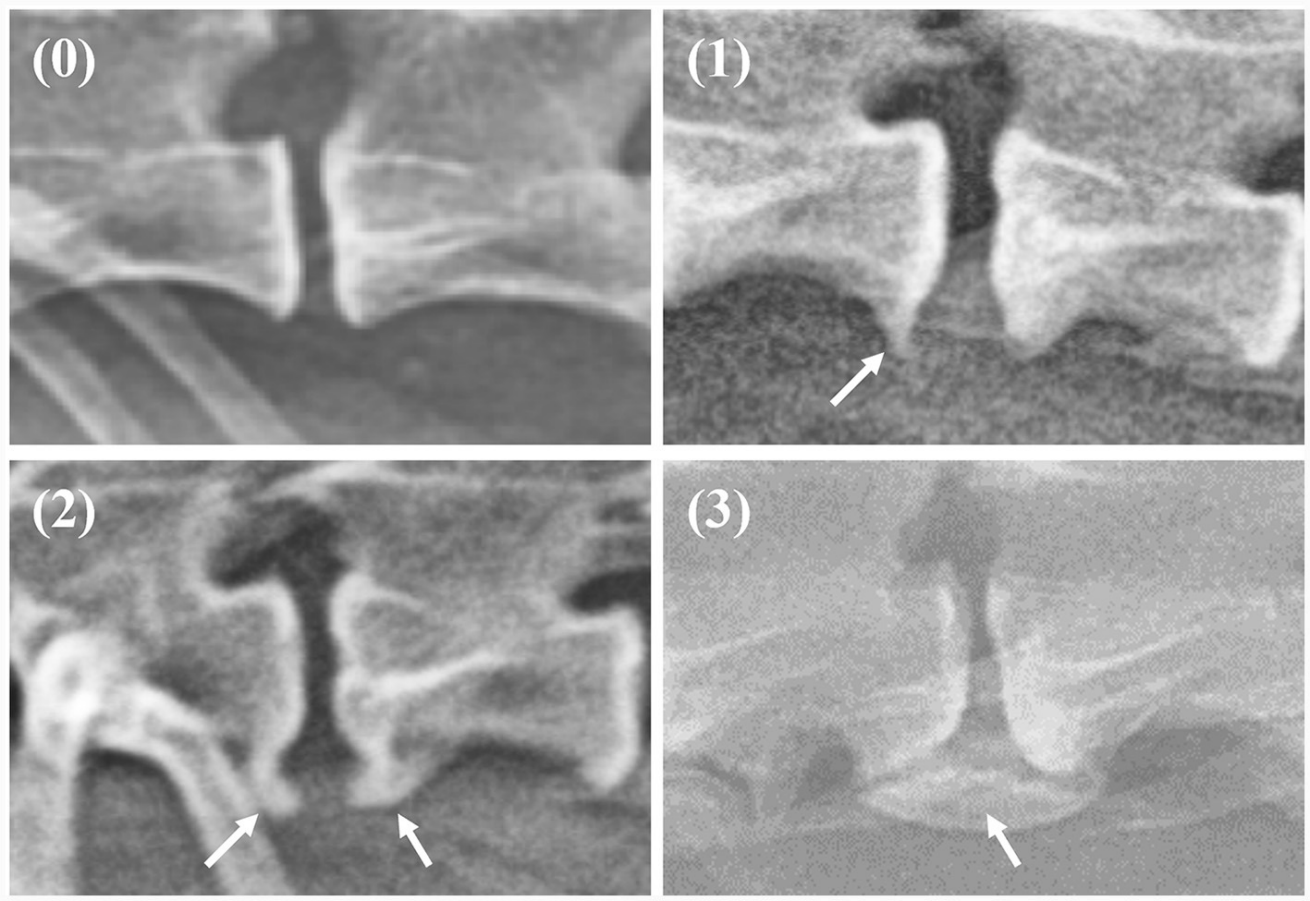
Grading of spondylosis deformans. Grade 0: no bony spur at the vertebral endplate; Grade 1: small bony spur at the edge of the vertebral endplate not extending past the endplate; Grade 2: more developed bony spurs, but not connected to adjacent vertebra; Grade 3: bony spur connected to adjacent vertebra forming bony bridges.

On thoracolumbar lateral radiographs, the vertebral body from T10 to L3 was evaluated, while on lumbar lateral radiographs, the vertebral body from L1 to L7 was evaluated. Spondylosis deformans was classified into three grades, as shown in Figure 1 (1, 6, 20). The area where spondylosis deformans occurred was checked, and the most frequently affected area was evaluated.

### 2.3 Deep learning model development

#### 2.3.1 Manual segmentation

**Figure 2:**
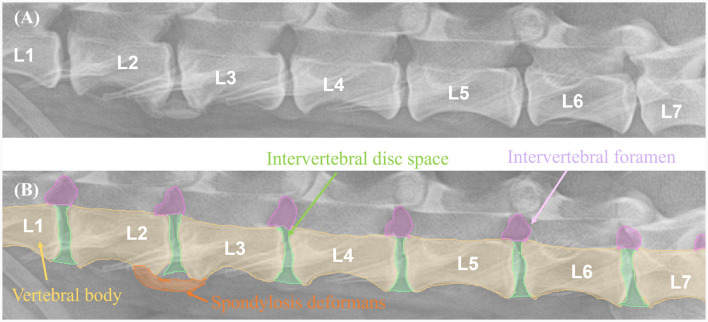
Example of manual segmentations. In the thoracolumbar lateral X-ray images **(A)**, the vertebral body (yellow), intervertebral disc space (green), intervertebral foramen (pink), and spondylosis deformans (orange) are labeled with separate colors using a segmentation tool (MediLabel software) to distinguish them **(B)**.

The X-ray images used in this study were manually labeled by 13 veterinary clinicians (residents in the Veterinary Medical Imaging Department of the Teaching Hospital of Jeonbuk National University) using MediLabel software (Ingradient, Inc., Seoul, South Korea). In thoracolumbar and lumbar lateral X-ray images, separate colors were used for labeling to distinguish spondylosis deformans, vertebral bodies, intervertebral disc space, and intervertebral foramen. To label the areas of spondylosis deformans, two veterinarians analyzed the radiographs and selected areas of common agreement. Figure 2 shows an example of manual segmentation of a lumbar lateral X-ray image.

#### 2.3.2 Data preprocessing

**Figure 3:**
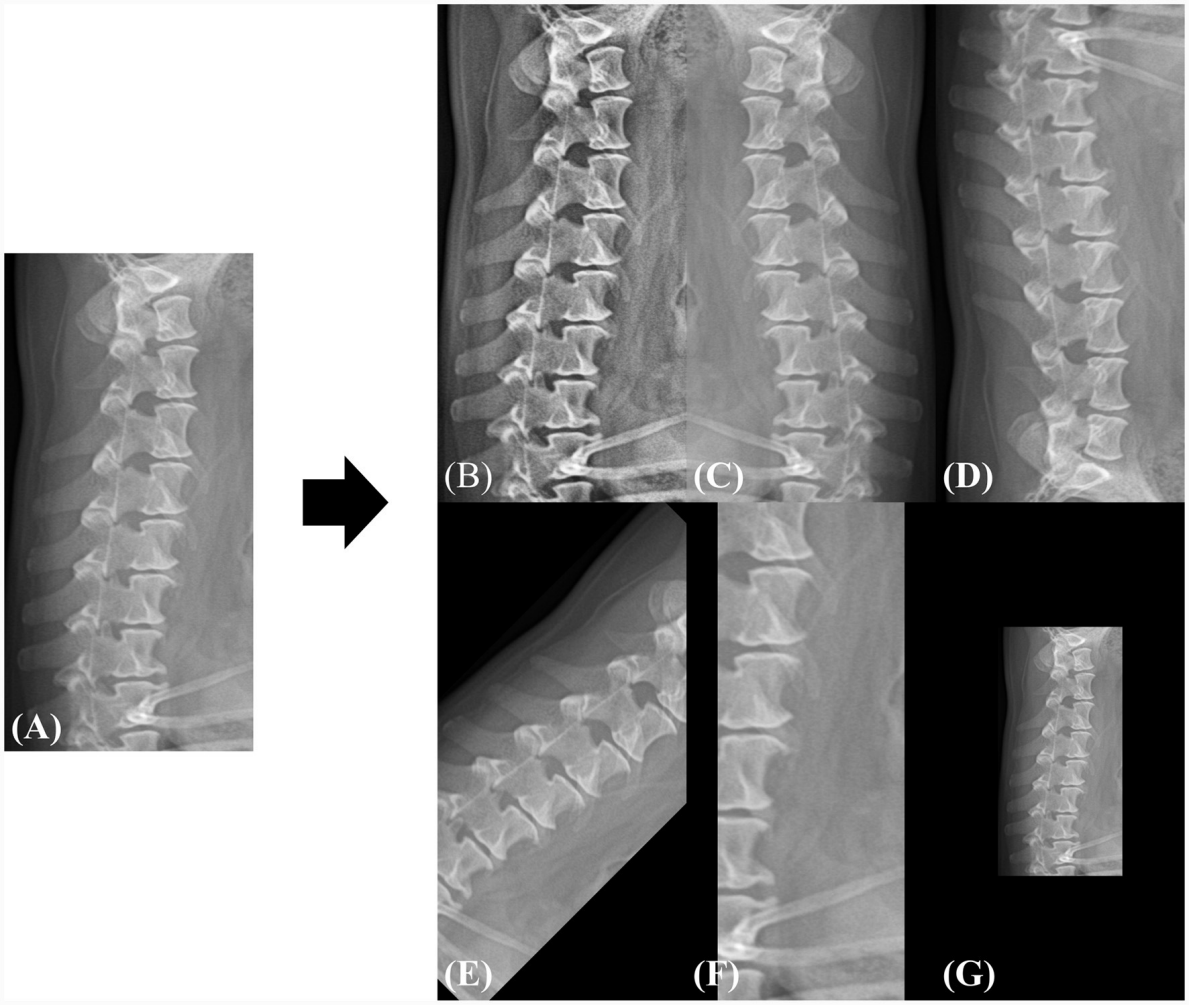
Example of data augmentation during the training phase. **(A)** The original image is randomly augmented by **(B)** adaptive histogram equalization, **(C)** horizontal flip, **(D)** vertical flip, **(E)** rotation, **(F)** zoom-in, and **(G)** zoom-out. All spatial augmentations were performed using cropping or zero-padding to match the original dimensions. The rotation angle was randomly selected between −45 and 45 and the zoom range was selected between 0.5 × and 1.5 × of the original dimensions.

Owing to the varying resolutions of the dataset used in this study, all images were resized to a uniform dimension, and the subsequent predictions were interpolated back to their original resolutions. To maintain the aspect ratio and detailed information of the images, we set the input resolution to 1024 pixels in height and 512 pixels in width. To increase data diversity and robustness, the images were augmented by flipping (horizontal, vertical), rotation, adaptive histogram equalization (21), zooming in with random cropping, and zooming out with a zero pad. Each augmentation was utilized during the training phase with a probability of 0.5. Figure 3 shows an example of the augmented samples. In addition, the image intensity was normalized into the range of 0 to 1 as shown in Eq. 1, where *I* is the original image, and *I*_max_ and *I*_min_ represent the maximum and minimum values of the image intensity, respectively.


(1) $$\begin{array}{l} I_{norm}=\;\frac{I-I_{\min}}{I_{\max}-I_{\min}} \end{array}$$

#### 2.3.3 Network architecture

**Figure 4:**
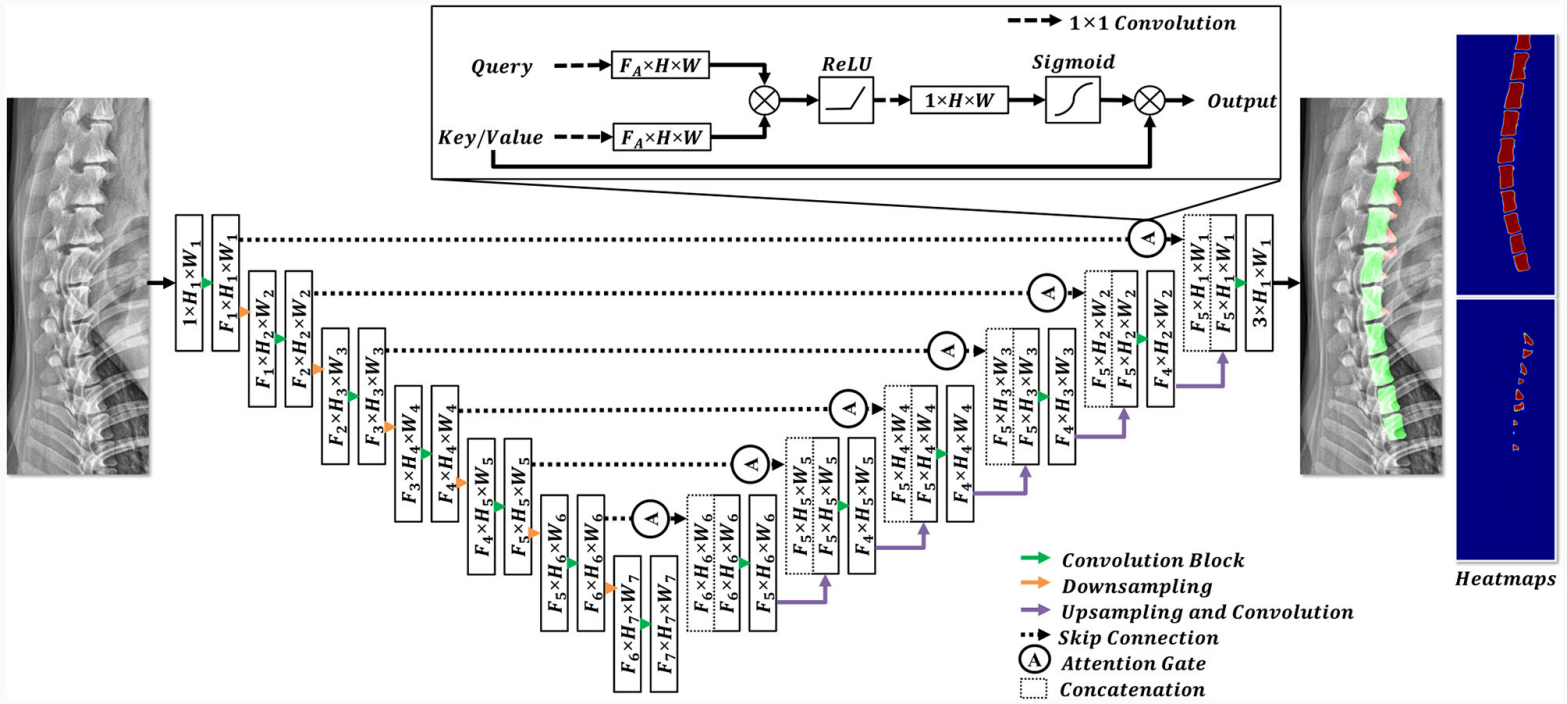
Structural diagram of the employed deep learning model. In the encoding stage, the features were downsampled (indicated by the orange arrow), whereas the feature dimensions were expanded using convolution blocks (highlighted by the green arrow). Each convolution block comprised a 3 × 3 convolution and an activation function. In the decoding stage, the features from the encoding stage were concatenated with the upsampled features after the implementation of a skip connection and an attention gate. Subsequently, the features were sequentially upsampled to their original resolutions. The final prediction included three channels, each representing a different pixel class.

In this study, a convolutional neural network (CNN) segmentation network was employed (Figure 4). The architecture of this model is based on attention U-Net (22), which has shown promising results in medical image segmentation tasks. To cope with the large variance in dog size (range: 1.64 kg−36 kg), the modified attention U-Net in this study was designed to have deeper feature extraction (i.e., multiscale features) than the original attention U-Net (22) architecture. The designed network extracts features at 7 levels, reducing the spatial resolution from (1024, 512) to (8, 16) for height and width, respectively. The filter dimensions of the model (*F*_1_, *F*_2_, *F*_3_, *F*_4_, *F*_5_, *F*_6_, *F*_7_) were selected as 16, 32, 64, 128, 256, 512, and 1024, respectively. The attention gate introduced in the attention U-Net retained in its original structure (22).

#### 2.3.4 Loss function and implementation details

Recent studies proposed various loss functions for successful segmentation under various conditions (23, 24). To successfully train the designed model to simultaneously segment the vertebral bodies and detect spondylosis deformans, we utilized a combination of two loss functions. The utilized loss function consists of a region-based loss function and a distribution-based loss function (23, 24). The region-based loss function allows the deep learning model to successfully segment the vertebral bodies from images, and the objective of the distribution-based loss function is to create a deep learning model to detect spondylosis deformans pixel-wise. The weighted categorical cross-entropy loss (25) and focal Tversky loss function (26) were used. The total loss function is expressed as follows:


$$\begin{array}{l} TI\left(y,ŷ\right)=\frac{1+yŷ}{1+yŷ+\beta \left(1-y\right)ŷ+\left(1-\beta \right)y\left(1-ŷ\right)}\\ {\;L}_{FTL}\left(ŷ,\;y\right)=\overset{3}{\underset{c=1}{\sum }}{\left(1-TI_{c}\right)}^{\gamma }\\ {\;L}_{WCE}\left(ŷ,\;y\right)=-\overset{3}{\underset{c=1}{\sum }}w^{\left(c\right)}\times y^{\left(c\right)}\log{ŷ}^{\left(c\right)}\\ {\;L}_{TOTAL}\left(ŷ,\;y\right)=\;L_{WCE}\left(ŷ,\;y\right)+L_{FTL}\left(ŷ,\;y\right) \end{array}$$

where *y*, ŷ represent the prediction and ground-truth probability map, *c* is the corresponding class (i.e., background, normal vertebral body, spondylosis deformans), *w* is the class weighting factor, and β, γ are hyperparameters. The *w* was set to 1, 1, and 5 for the background, normal vertebral body, and spondylosis deformans. β, γ were set at 0.3 and 0.75. The weighting factors and hyperparameters were determined empirically to obtain the best results. To optimize the utilized loss function, the Adam optimiser (27) with a learning rate of 1*e*^−4^ was used, and early stopping criteria were used to obtain the best result. The proposed methods were implemented using MONAI (28) and PyTorch (29) frameworks.

### 2.4 Time measurement

For the 25 lateral thoracolumbar and lumbar X-ray images used as validation data, the time required per image to detect spondylosis deformans by a veterinary clinician and by the deep learning model through auto-segmentation was recorded and compared.

### 2.5 Model accuracy and statistical analysis

Dice similarity coefficient (DSC) was used to confirm whether auto-segmentation and manual segmentation were in close agreement for the vertebral body, intervertebral disc space, and foramen. The DSC is a relative measure of the percentage of pixels that overlap between auto-segmentation and manual segmentation images (30). The closer the DSC is to 1, the better the match between the two segmentations. DSC was performed using the following equation (30):


$$\begin{array}{l} \text{DSC }=\;2\;\left(\text{Intersected region}\right)/\left(\text{sum of region segmentations}\right) \end{array}$$

To evaluate how closely matched spondylosis deformans were detected by the deep learning model and veterinary clinicians, Cohen's kappa analysis was performed to check sensitivity and specificity. Additionally, Cohen's kappa test was used to determine inter-veterinarian agreement on the areas determined to have spondylosis deformans. Cohen's kappa results were interpreted as follow: values ≤ 0.00–0.20 indicated non- to slight, 0.21–0.40 indicated fair, 0.40–0.60 indicated moderate, 0.60–0.80 indicated substantial, and 0.80–1.00 indicated almost perfect agreement (31).

To identify the relationship between the presence of spondylosis deformans and the occurrence of thoracolumbar disc disease-related neurologic signs, a Chi-square test was performed. The values were determined to be statistically significant at *p* < 0.05.

SPSS version 29.0 (SPSS Corp., Armonk, NY, USA) was used for statistical analyses.

## 3 Results

### 3.1 Animals

A total of 29 breeds were enrolled in the study: Maltese (*n* = 39), Pomeranian (*n* = 18), Poodle (*n* = 16), Dachshund (*n* = 13), mixed breed (*n* = 11), Shih Tzu (*n* = 9), Pekingese (*n* = 9), Cocker Spaniel (*n* = 8), Miniature Poodle (*n* = 5), Chihuahua (*n* = 4), Bichon Frise (*n* = 4), Beagle (*n* = 3), Yorkshire Terrier (*n* = 3), Golden Retriever (*n* = 2), German Shepherd (*n* = 2), Old English Sheepdog (*n* = 2), Jindo (*n* = 2), Boston Terrier (*n* = 1), Labrador Retriever (*n* = 1), Miniature Pinscher (*n* = 1), Pompitz (*n* = 1), Samoyed (*n* = 1), Siberian Husky (*n* = 1), Schnauzer (*n* = 1), Shetland Sheepdog (*n* = 1), Spitz (*n* = 1), Welsh Corgi (*n* = 1), Sapsaree (*n* = 1), and Whippet (*n* = 1). The average weight was 7.42 kg (range: 1.64–36 kg), and the average age was 8.81 years (range: 0.7–17 years), and for seven dogs, there were no information regarding body weight. This study included 91 male dogs (24 intact, 67 castrated) and 71 female dogs (20 intact, 51 spayed).

### 3.2 Deep learning model shows considerable similarity compared to manual segmentation in recognizing vertebral body

**Figure 5:**
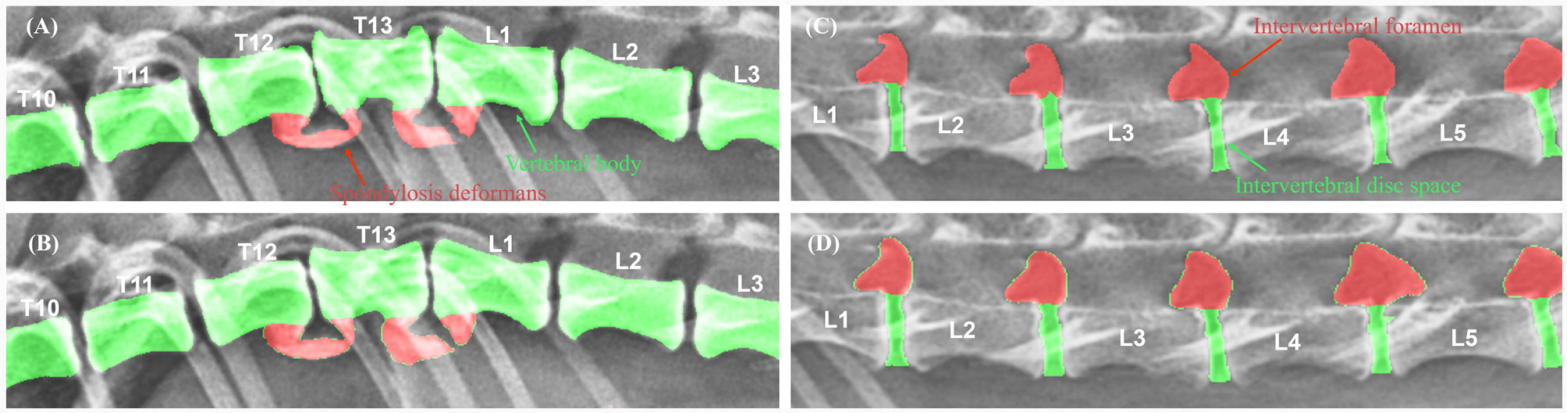
Examples of manual vs. automated segmentation of vertebrae, intervertebral disc space, and foramen. Results of manual segmentation **(A, C)** of vertebral body, spondylosis deformans, intervertebral disc space and intervertebral foramen; automatic segmentation **(B, D)** in thoracolumbar and lumbar lateral radiographs.

To evaluate the performance of the model, the DSC between manual and automated segmentation was calculated for a validation dataset of 25 dogs. Post-processing, the average DSC value for the vertebral body was 0.910 ± 0.038 (mean ± SD). The DSC values of the intervertebral disc space and foramen were 0.787 ± 0.056 and 0.779 ± 0.083, respectively. Figure 5 shows an example of manual and automated segmentation of the vertebral body, spondylosis deformans, intervertebral disc space, and intervertebral foramen.

### 3.3 Deep learning model shows a high capability of detecting spondylosis deformans in a short time

The interclass correlation kappa value between veterinary clinicians for the evaluation of spondylosis deformans in thoracolumbar and lumbar lateral X-ray images was 0.889, confirming almost perfect agreement. In addition, when the deep learning model recognized spondylosis deformans on thoracolumbar and lumbar lateral X-ray images in validation dataset, the kappa value with identification by a veterinary clinician was 0.813, indicating an almost perfect agreement. The sensitivity was 87.5%, specificity was 98.5%, positive predictive rate was 77.8%, and negative predictive rate was 99.3% (Table 1). Upon checking the ability of the deep learning model on abdominal lateral X-ray images in the test dataset, the kappa value was 0.839, indicating an almost perfect agreement. The sensitivity was 98.4 %, specificity was 86.3%, positive predictive rate was 86.1%, and negative predictive rate was 98.4% (Table 1). When we checked how well deep learning recognized 63 sites with spondylosis deformans identified by veterinary clinicians in the test dataset, it detected spondylosis deformans in 96.2% of the cases in grade 1 and 100% of the cases in grades 2 and 3 (Table 2).

**Table 1:** Cohen's kappa analysis between a veterinary clinician and deep learning model for detection of spondylosis deformans.

	**Kappa value**	**Sensitivity (%)**	**Specificity (%)**	**PPV (%)**	**NPV (%)**
Validation dataset	0.813^*^	87.5	98.5	77.8	99.3
Test dataset	0.839^*^	98.4	86.3	86.1	98.4

95% CI, 95% confidence interval; PPV, positive predictive value; NPV, negative predictive value.

Experimental values were considered significant at *p* < 0.01^*^.

**Table 2:** Detection rate based on the grade of spondylosis deformans by deep learning.

**Grade**	**Sensitivity (%)**	**No. of detected spondylosis deformans by deep learning**	**Total no. of spondylosis deformans**
1	96.2	25	26
2	100	26	26
3	100	11	11

The relationship between spondylosis deformans and clinical signs associated with thoracolumbar and lumbar disc disease was examed in 152 dogs. If a dog displayed multiple grades of spondylosis deformans, we selected the highest grade lesion for comparison. In this study, ~33.3% of dogs without spondylosis deformans, 45.8% with grade 1, 47.1% with grade 2, and 46.7% with grade 3 exhibited clinical signs associated with thoracolumbar and lumbar disc disease (Table 3).

**Table 3:** Relationship of the presence or grade of spondylosis and clinical signs related to the disc disease in 152 dogs.

	**Grade 0**	**Grade 1**	**Grade 2**	**Grade 3**	**Total**
No. of dogs with neurologic signs	32 (21.1%)	11 (7.2%)	8 (5.3%)	7 (4.6%)	58 (38.2%)
No. of dogs not presenting neurologic signs	64 (42.1%)	13 (8.6%)	9 (5.9%)	8 (5.3%)	94 (61.8%)
Total	96 (63.1%)	24 (15.8%)	17 (11.2%)	15 (9.9%)	152 (100%)

The mean time taken by deep learning to automatically segment vertebral bodies and detect spondylosis deformans was found to be 0.052 s per image, while the time taken by a veterinary clinician to evaluate spondylosis deformans was found to be 17.92 s per image.

### 3.4 Spondylosis deformans was more common at T12-T13 and L2-L3 in thoracolumbar and lumbar X-ray images

**Figure 6:**
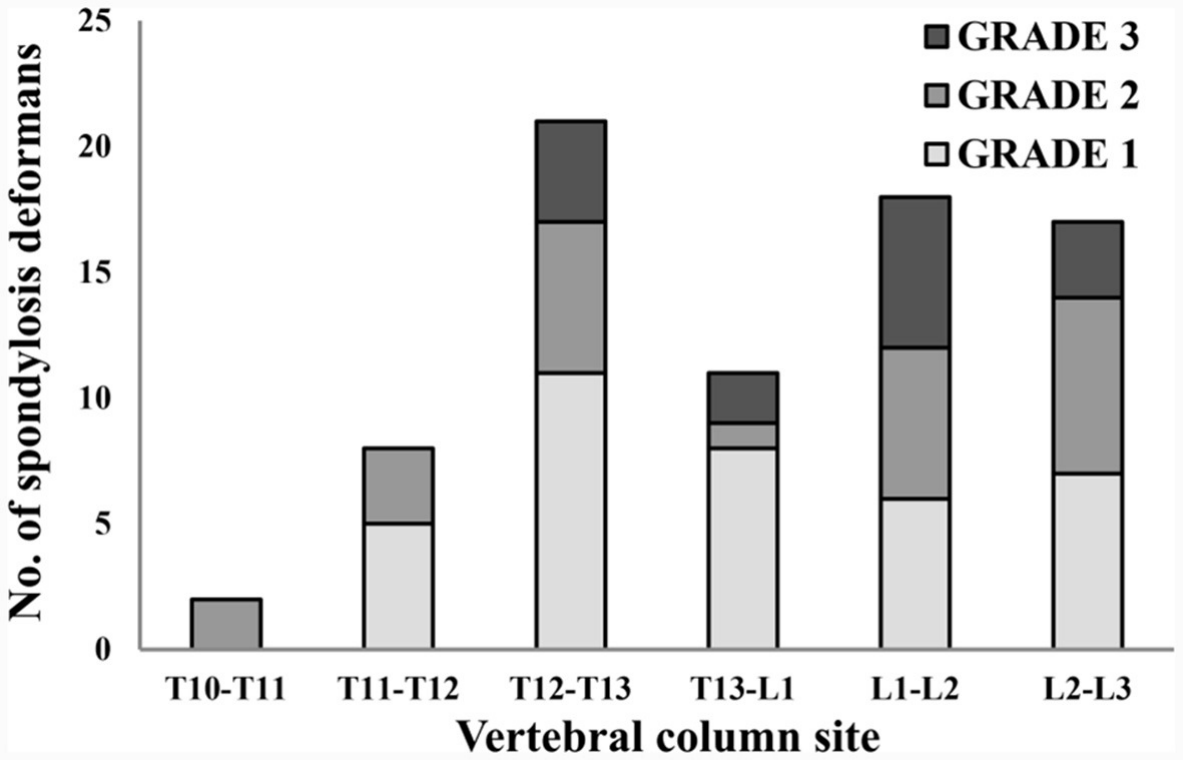
Distribution of sites for spondylosis deformans and grading of dogs in 119 thoracolumbar lateral X-ray images. T, thoracic; L, lumbar.

**Figure 7:**
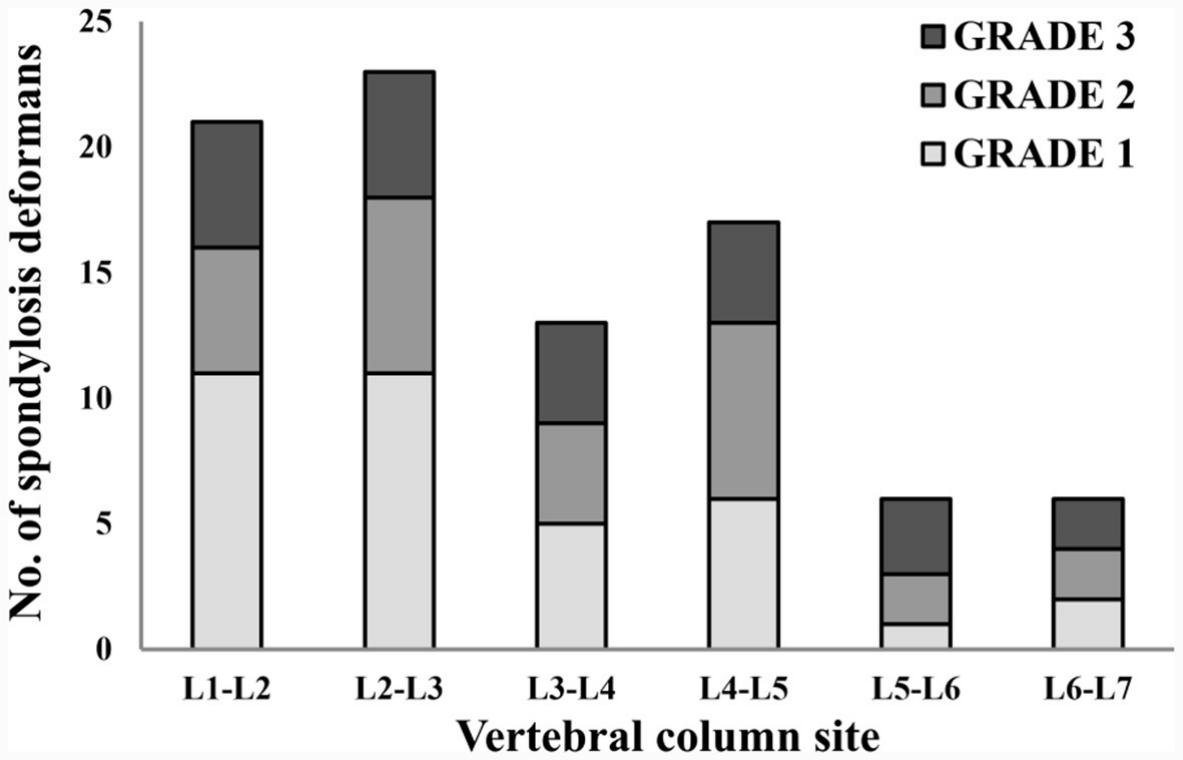
Distribution of sites for spondylosis deformans and grading of dogs in 146 lumbar lateral X-ray images. L, lumbar.

A total of 265 thoracolumbar and lumbar lateral radiographs of dogs were evaluated for spondylosis deformans. Of these, 163 dogs exhibited spondylosis deformans (thoracolumbar vertebrae: 77 sites, lumbar vertebrae: 86 sites), of which 30 (18.4%) had dorsal spondylosis deformans (Tables 4, 5). Within the thoracolumbar vertebrae (T10-L3), the most commonly affected sites included T12-T13 (36.7%) (Figure 6). In the lumbar vertebrae (L1-L7), the most commonly affected sites were L2-L3 (26.7%) (Figure 7). In contrast, T10-T11 (2.6%), L5-L6 (7.0%), and L6-L7 (7.0%) were identified as the least affected areas.

**Table 4:** Number of spondylsis deformans cases and grading of dogs in 119 thoracolumbar lateral X-ray images.

	**T10-T11**	**T11-T12**	**T12-T13**	**T13-L1**	**L1-L2**	**L2-L3**
Grade 1	0 (0)	5 (0)	11 (0)	8 (0)	6 (2)	7 (0)
Grade 2	2 (0)	3 (1)	6 (0)	1 (0)	6 (1)	7 (0)
Grade 3	0 (0)	0 (0)	4 (1)	2 (0)	6 (1)	3 (1)

T, thoracic; L, lumbar; Numbers in parentheses indicate number of dorsal spondylosis deformans cases.

**Table 5:** Number of spondylsis deformans cases and grading of dogs in 146 lumbar lateral X-ray images.

	**L1-L2**	**L2-L3**	**L3-L4**	**L4-L5**	**L5-L6**	**L6-L7**
Grade 1	11 (3)	11 (0)	5 (1)	6 (3)	1 (1)	2 (2)
Grade 2	5 (1)	7 (0)	4 (2)	7 (3)	2 (1)	2 (1)
Grade 3	5 (0)	5 (2)	4 (2)	4 (1)	3 (0)	2 (0)

L, lumbar; Numbers in parentheses indicate number of dorsal spondylosis deformans cases.

## 4 Discussion

This study is the first to develop deep learning models to automatically segment the vertebral body and detect spondylosis deformans in the thoracolumbar and lumbar lateral radiographs of dogs. Recently, models to detect specific diseases in X-ray images of dogs have been developed and commercialized, and studies have been conducted to auto-segment gross head and neck tumors for radiation therapy and automatically detect kidney calculi and measure kidney volume in CT images in dogs; however, there have been no studies in the literature on a deep learning model to automatically recognize disc diseases in X-ray images of dogs (32–34).

First, a segmentation model was developed to automatically detect the vertebrae and disc space and was then compared to deep learning models developed for lumbar vertebrae detection in prior human literature. The mean intersection over union value, a term similar to DSC for vertebral bodies, was found to be 0.8–0.88 in a human study, and when the DSC value of the vertebral body (0.910) in this study was converted to the mean intersection over union value, it was found to be 0.835, showing the ability to recognize the vertebral body at a similar level to the previous human research (9).

However, in this study, the DSC for the intervertebral disc space and intervertebral foramen were slightly lower, reaching up to 0.787 and 0.779 respectively. The DSC value for the intervertebral disc space and foramen were unavailable in the aforementioned human study, which is likely because the size of the disc space and intervertebral foramen is relatively smaller than that of the vertebral bodies. Inconsistent imaging of the vertebral endplates can cause disc space distances to appear shorter than they actually are or results in unclear margins. Moreover, intervertebral foramen may have ambiguous borders with adjacent structures. Additionally, the substantial relative weight difference between breeds in dogs compared to humans might have also affected this value. We assume that training with a larger number of images will be required to improve the recognition rate of disc spaces and intervertebral foramen.

In this study, the most affected areas of spondylosis deformans in thoracolumbar and lumbar lateral radiographs of dogs were identified as T12-T13 and L2-L3, with T12-L5 being more affected overall. Previous studies (3, 4, 35) have found that the most affected area was somewhat different (L1-L3); however, the overall commonality of occurrence in the vertebral region (T12-L5) was similarly confirmed.

Of the total 163 cases of spondylosis deformans identified in 265 animals, 30 cases of dorsal spondylosis deformans (18.4%) were identified. However, of the total 63 spondylosis deformans in the test dataset, only 3 cases of dorsal spondylosis deformans (2 sites for grade 1 and 1 site for grade 2) were identified, but the deep learning model accurately recognized all of them as spondylosis deformans. Although the number of images with dorsal spondylosis deformans in the test dataset is insufficient to evaluate accuracy, in the training and validation datasets, the deep learning model also demonstrated accurate recognition in the training and validation datasets, identifying 26 out of 27 dorsal spondylosis deformans. This suggests a high likelihood of accurate recognition for dorsal spondylosis deformans.

Even for very small lesions such as grade 1 spondylosis deformans, the model successfully detected 25 out of 26 sites, achieving a remarkably high sensitivity of 96.2% and a very high negative predictive value of 98.4%. The specificity and positive predictive values were also high, at 86.3% and 86.1%, respectively. These results suggest that the deep learning model in this study exhibited higher sensitivity toward very small spondylosis deformans than two clinicians. This may likely be attributed to the criteria of the model, which that considered a site as true spondylosis deformans only if both clinicians identified it as spondylosis deformans. Notably, when we identified 10 sites in our test dataset as spondylosis deformans using the deep learning model alone, 6 of them were sites judged as spondylosis deformans by one of the two clinicians.

In addition, the time taken by the deep learning model to auto-segment spondylosis deformans in radiographic images was significantly faster than that by a veterinary clinician to visually evaluate them, which we believe could ultimately save the interpretation time of veterinary clinicians.

In case of the test dataset, images of the vertebral region cropped from the lateral abdominal X-ray were used, when checked ability of the deep learning model to detect spondylosis deformans, observed that the sensitivity, specificity, positive predictive rate, and negative predictive rate were all higher than 86%. These results mean that the deep learning model can detect spondylosis deformans highly accurately not only in thoracolumbar and lumbar lateral X-ray images taken with precise vertebral endplate alignment but also in lateral abdominal X-ray images taken routinely for general medical examinations, which is expected to be useful in veterinary clinical practice. However, when applying the deep learning model to uncropped plane lateral abdominal X-ray images, detection capability of vertebral bodies and spondylosis deformans is slightly lower than applying cropped images. Therefore, additional advancements training are considered necessary to fully apply it to general plane lateral abdominal X-ray images.

When the association between the presence of spondylosis deformans and thoracolumbar and lumbar disc disease-related clinical signs was examined, no statistical significance was found. However, dogs with spondylosis deformans were more likely to exhibit clinical signs associated with disc disease. However, no association was found between clinical signs and the grade of spondylosis deformans, and the presence of spondylosis deformans did not differ by breed in this study. Previous studies also have similarly shown that spondylosis deformans is detected on radiographic images in 17.8%−32.8% of normal dogs without specific clinical signs related to disc diseases (35–37). However a higher rate of prevalence of spondylosis deformans (~47%) is identified in patients with intervertebral disc protrusion, a form of intervertebral disc disease (IVDD) (38). Hence, while the presence of spondylosis deformans is not specific for IVDD, it can be useful in the diagnosis of disc disease when considered in conjunction with radiologic findings such as intervertebral space narrowing.

In conclusion, the deep learning model developed in this study is expected to help clinical veterinarians accurately and rapidly evaluate spondylosis deformans by determining sites of disc instability. Moreover, the vertebral body segmentation model developed in this study can be applied to develop deep learning models that automatically detect vertebral body diseases such as tumors, discospondylitis, dislocation of vertebrae, and abnormal narrowed intervertebral disc space.

## Data availability statement

The raw data supporting the conclusions of this article will be made available by the authors, without undue reservation.

## Ethics statement

The animal studies were approved by the Institutional Animal Care and Use Committee of Jeonbuk National University (approval nos. JBNU NON2022-085 and NON2023-023). The studies were conducted in accordance with the local legislation and institutional requirements. Written informed consent was obtained from the owners for the participation of their animals in this study.

## Author contributions

JP: Conceptualization, Data curation, Investigation, Writing – original draft, Formal analysis, Methodology, Validation, Writing – review & editing. HC: Conceptualization, Investigation, Methodology, Writing – original draft, Writing – review & editing, Data curation, Formal analysis, Validation. YJ: Conceptualization, Methodology, Writing – review & editing, Investigation. KL: Conceptualization, Project administration, Writing – review & editing, Supervision. HY: Conceptualization, Data curation, Investigation, Project administration, Supervision, Writing – review & editing, Methodology, Validation, Writing – original draft.
